# A Case of Rupture of the Uterus, Which Terminated Favourably

**Published:** 1816-06

**Authors:** 


*For the London Medical and Physical Journal.*

### *A Case of Rupture of the Uterus, which terminated favourably;* by J. W., of Maidenhead, Berks.

RUPTURES of the Uterus so generally terminate in death, that I believe there are many, even of the present enlightened generation, who never knew, or can scarcely believe, it terminate in recovery. For proof that such cases, however, have occurred, we can refer to Dr. Douglas's Account of Mrs. Manning, and Dr. Hamilton's Case in his Outlines of Midwifery. The truth of the following may be relied on.

March 29th.—Was desired to visit Mrs. W., of a relaxed habit of body, about twenty-eight years of age, and in the seventh month of her pregnancy.

Nothing material had occurred during the former part of her pregnancy, but a day or two previous to my seeing her a very profuse hæmorrhage had taken place, and she had slight pains in the region of the uterus.

30th.—The pain very materially increased; the hæmorrhage had been very inconsiderable. A glyster was thrown up, which produced a sufficient evacuation; and the following mixture was prescribed:

℞. Nitrat. Potassæ.

Pulv. Tragac. Compos. ā ʒij.

Aq. Puræ, ℥vj.

Tincturæ Opii, ʒifs. f. M.

The pain continued very violent, and towards the evening began to bear down. Upon examination, I could not discover the os internum the least dilated.

April 1st.—On examining, at one o'clock in the morning, I found the membranes ruptured, and the os internum so much dilated, that I clearly discovered the presentation of the shoulder: the hand and arm being situated behind the child. The patient appearing much exhausted, and her attendants extremely anxious for her safety, I solicited the assistance of a physician, but, before his arrival, endeavoured (during an interval of pain) to bring the arm forward, in order to prosecute the turn with the greater facility, which was accomplished much sooner, and with greater success, than I expected. The fœtus was highly putrid, and, from appearances, had been some time dead. Waiting in vain for a pain to assist in extracting the placenta, I was forced to introduce my hand into the uterus (as the funis was perfectly rotten), and withdraw the greater part of it. On the second attempt to bring away the remainder, I discovered a very alarming laceration, through the posterior and inferior part of of the uterus. The doctor now entered the room, and, on examining, expressed his surprise at feeling distinctly the intestines and their convolutions.

April 2d.—The patient much better than I expected, notwithstanding severe pains about the uterus and abdomen, which I was pleased to find alleviated by administering the following enema:

℞. Sacchar. non Purif. ℥iiſs.

Ol. Oliv. Opt. ℥iſs.

Lact. Vaccini ℥x. M. statim. infundend.

She afterwards took a mixture of nitre and opium.

The third day after delivery, the pain great; the discharge highly tinctured with blood. On the fourth day the pain very little; the discharge trifling.—Cont. medicam. ut ante.

The fifth day, entirely free from pain. From that time, altered her plan of regimen, when she every day recovered her strength, and at the end of three weeks pursued her usual domestic employ.

